# Danthron, an Anthraquinone Isolated from a Marine Fungus, Is a New Inhibitor of Angiogenesis Exhibiting Interesting Antitumor and Antioxidant Properties

**DOI:** 10.3390/antiox12051101

**Published:** 2023-05-15

**Authors:** Isabel Vidal, José Antonio Torres-Vargas, José María Sánchez, Mónica Trigal, Melissa García-Caballero, Miguel Ángel Medina, Ana R. Quesada

**Affiliations:** 1 Department of Molecular Biology and Biochemistry, Faculty of Sciences, University of Málaga, Andalucía Tech, and IBIMA Plataforma BIONAND, E-29071 Málaga, Spain; isabel25.vidal@gmail.com (I.V.); torresvargas@uma.es (J.A.T.-V.); melissa@uma.es (M.G.-C.); medina@uma.es (M.Á.M.); 2 Biomar Microbial Technologies, Parque Tecnológico de León, Parcela M-10.4, Armunia, 24009 León, Spain; jm.sanchez@biomarmt.com (J.M.S.); m.trigal@biomarmt.com (M.T.); 3 Unidad 741 de CIBER “de Enfermedades Raras”, E-29071 Málaga, Spain

**Keywords:** danthron, anthraquinone, antiangiogenic, angioprevention, antitumor, antioxidant, marine fungus, cancer, angiogenesis

## Abstract

The role played by a sustained angiogenesis in cancer and other diseases stimulates the interest in the search for new antiangiogenic drugs. In this manuscript, we provide evidence that 1,8- dihydroxy-9,10-anthraquinone (danthron), isolated from the fermentation broth of the marine fungus *Chromolaenicola* sp. (HL-114-33-R04), is a new inhibitor of angiogenesis. The results obtained with the in vivo CAM assay indicate that danthron is a potent antiangiogenic compound. In vitro studies with human umbilical endothelial cells (HUVEC) reveal that this anthraquinone inhibits certain key functions of activated endothelial cells, including proliferation, proteolytic and invasive capabilities and tube formation. In vitro studies with human breast carcinoma MDA-MB231 and fibrosarcoma HT1080 cell lines suggest a moderate antitumor and antimetastatic activity of this compound. Antioxidant properties of danthron are evidenced by the observation that it reduces the intracellular reactive oxygen species production and increases the amount of intracellular sulfhydryl groups in endothelial and tumor cells. These results support a putative role of danthron as a new antiangiogenic drug with potential application in the treatment and angioprevention of cancer and other angiogenesis-dependent diseases.

## 1. Introduction

Angiogenesis, the formation of new blood vessels from a pre-existing vascular bed, is a highly regulated process that in adulthood is activated only in certain physiological situations, such as wound healing, bone fracture recovery and other processes which are mainly related to woman reproductive cycles. On the contrary, a persistently activated angiogenesis is observed in several diseases, including wet macular degeneration, some retinopathies, psoriasis, diabetes, rheumatoid arthritis and cancer [1], making the inhibition of angiogenesis a field of utmost interest in pharmacology [2]. Undoubtedly, the greatest enthusiasm in antiangiogenic therapies derives from their application in oncology, since angiogenesis, one of the hallmarks of cancer, is crucial to tumor growth, invasion and metastasis [3], and cancer is now the leading cause of premature death in developed countries [4]. In fact, the number of FDA-approved antiangiogenic therapies for cancer is continuously increasing [5]. In addition, with an unstoppable increase in the number of new cancer cases, many of them being detected in early stages, the health care costs derived from the longer duration of their treatment makes it necessary to invest efforts in the development of cancer preventive strategies [4]. Since angiogenesis inhibition may lead to slowing down the progression of the tumor at different stages, angiopreventive strategies may be applied to any of the three levels of chemoprevention: primary angioprevention, focused on the general population and based on nutritional recommendations; secondary angioprevention, devoted to those who are at high-risk for developing cancer and could benefit from long-term schedules based on the use of antiangiogenic nutraceuticals; and tertiary angioprevention in which cancer patients could improve their treatments and increase disease-free survival thanks to antiangiogenic drugs [6].

Our group is actively involved in the search for new drug candidates which could be used for the treatment of cancer and other angiogenesis-dependent diseases [7,8,9,10]. In this context, the identification of new inhibitors of angiogenesis from marine origin is of highest interest [11,12,13,14].

Marine organisms have a huge and still unexplored potential for the production of bioactive molecules. Indeed, some of them have been approved as anticancer drugs [15]. In many cases, those compounds are produced by symbiotic microorganisms which are prolific providers of a wide diversity of secondary metabolites conferring several evolutionary advantages, including mechanisms of defense against potential predators [16]. Marine-derived fungi are a rich source of bioactive compounds with both chemical and biological diversities [17]. Among fungal secondary metabolites, anthraquinones, in particular 1,8- dihydroxy-anthraquinones, are some of the most studied compounds for their known pharmacological activity [18]. In fact, more than 200 anthraquinones and structurally related compounds have been isolated from different species of marine fungi [17].

**Figure 1:**
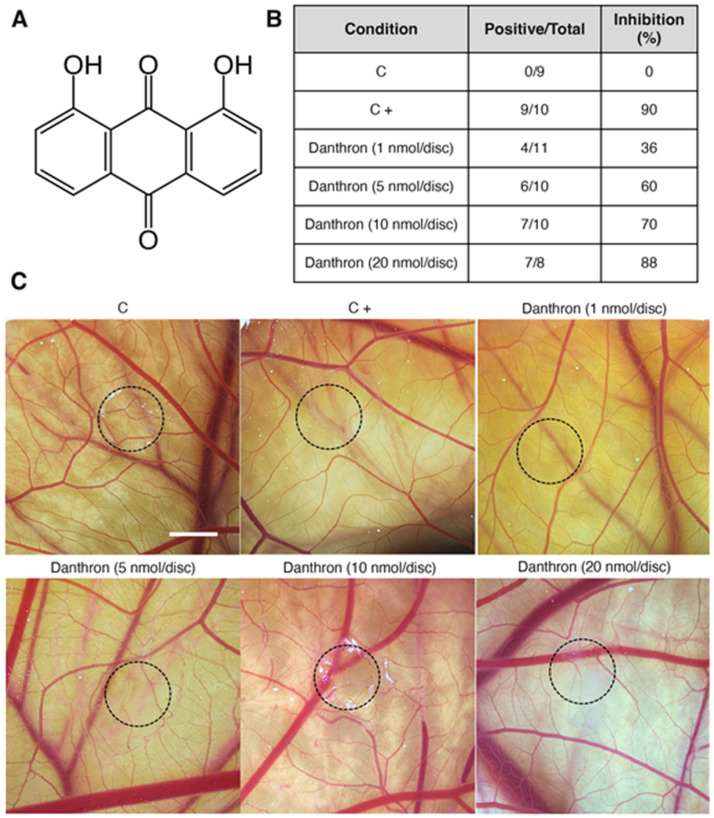
Chemical structure and antiangiogenic activity of danthron in vivo. (**A**) Chemical structure of danthron. (**B**) Table summarizing the chick chorioallantoic membrane (CAM) assay results. Number of positive CAMs (with impaired vasculature development) over total CAM number, and percentage of CAMs with inhibited angiogenesis for each treatment condition. (**C**) Representative photographs of CAMs with methylcellulose discs containing DMSO (C), aeroplysinin-1 (6 nmol/CAM, C+) or danthron at the indicated concentrations per CAM. Circles show the locations of the methylcellulose discs during treatment (scale bar = 2 mm).

In the course of a screening program, 1,8- dihydroxy-9,10-anthraquinone (danthron) (Figure 1A) was isolated and purified from the fermentation broth of a marine fungus belonging to the genus *Chromolaenicola* sp., based on its ability to inhibit endothelial cell differentiation in vitro [19]. The presence of danthron in marine sources is not uncommon, since it has also been reported to be produced by *Halorosellinia* sp. (No. 1403), *Guignardia* sp. (No. 4382) and *Beauveria bassiana* TPU942 marine fungi [20,21], in the Great Barrier Reef ascidian *Didemnum albopunctatum* [22] and in the bryozoan *Watersipora subtorquata* [23].

Interestingly, danthron can also be isolated from terrestrial sources, being the basic structure of the aglycones of naturally occurring laxative glycosides in several species of plants, including *Rheum* [24,25]. Rhubarb, the root and rhizome of *Rheum palmatum* L., is one of the oldest and best-known Chinese herbal medicines, and has been traditionally employed as a laxative, and for the treatment of constipation jaundice, gastrointestinal hemorrhage, renal fibrosis and ulcers. [26,27]. In fact, danthron was widely used from the beginning of the twentieth century as an over-the-counter laxative. In spite of some controversial data pointing to a putative carcinogenic activity of this and other anthraquinones, leading to a limitation of their current use in the treatment of constipation [28], the controlled use of danthron as a pharmaceutical has not been fully interrupted, being employed in some countries, including the United Kingdom [29]. Moreover, the interest in *Rheum palmatum* L. and its components has been recently re-awakened, on the basis of its anti-obesity and anti-psoriatic potential [30,31].

In this manuscript, we provide evidence that danthron is a new inhibitor of angiogenesis and give some clues about its mechanism of action. The antiangiogenic, antitumor and antioxidant activities of this compound suggest its potential usefulness for the treatment and angioprevention of cancer.

## 2. Materials and Methods

### 2.1. Materials

DMEM containing glucose (1 g/L and 4.5 g/L) and RPMI were purchased from Corning (Corning, NY, USA), while endothelial cell growth medium-2 (EGM-2) was obtained from LONZA (Basel, Switzerland). Fetal bovine serum (FBS) was obtained from BioWest (Kansas City, KS, USA). Trypsin, penicillin and streptomycin were purchased from BioWhittaker (Verviers, Belgium). Matrigel was obtained from Becton-Dickinson (Bedford, MA, USA). A solution of 30% acrylamide/bis (29:1) was purchased from BioRad (Hercules, CA, USA). Plastic material for cell culture was obtained from Nunc (Roskilde, Denmark). All other reagents not listed in this section were purchased from Merck Life Sciences Sigma Aldrich (Darmstadt, Germany).

### 2.2. Isolation of Danthron from the Fermentation Broth of Chromolaenicola sp. (HL-114-33-R04)

A 6 cm diameter chopped portion of a well-developed agar culture was used to inoculate 2 × 250 mL Erlenmeyer flasks with 40 mL of seeding medium each containing 2% oatmeal, 2% malt extract, 0.01% KH_2_PO_4_, 0.005% MgSO_4_ and cultured at 25 °C on a rotary shaker at 200 rpm. The flasks were incubated for 72 h in the dark, and used as a first stage inoculum. Erlenmeyer flasks (4 × 2 L) with 250 mL of medium each containing 2% cane sugar, 0.1% agar, 0.3% olive oil, 0.1% glycine, 0.3% KNO_3_ and 0.9% Dupla marine salt mixture adjusted to pH 6.7 were inoculated with 6% of the first-stage inoculum. The fermentation was carried out for seven days at 25 °C on a rotary shaker at 200 rpm in the dark. Production of *compound **1*** (danthron) was monitored by HPLC.

To a fermentation broth (1 L) of HL-114-33-R04 fungal strain, 200 mL of Amberlite XAD-1180N was added and stirred for 1 h. The mixture was filtered, and the exhausted supernatant discarded. The mycelium and the Amberlite-XAD-1180N were extracted with 1 L of a mixture of EtOAc/MeOH (3:1). The mixture was filtered and partitioned between EtOAc and water. The organic layer was allowed to dry, and the crude extract (1.5 g) was fractionated by flash chromatography on silica gel, eluting with a stepwise gradient of hexane/EtOAc. Fractions containing *compound **1*** (eluted with hexane/EtOAC 8:2) were finally purified by C18 reversed-phase column chromatography (MeOH/H_2_O, 9:1), affording 30 mg of *compound **1***.

*Compound **1*** was obtained as an orange amorphous solid and identified as danthron, an anthraquinone derivative, from spectroscopic data, and a comparison of these data was made with those of the reported values [32]: UV (MeOH) λmax 223, 252, 282 and 430 nm; 1H NMR (400 MHz, CDCl3) δ 7.26 (2H, dd, J = 8.5, 1.2 Hz), 7.65 (2H, dd, J = 8.5, 7.5 Hz), 7.78 (2H, dd, J = 7.5, 1.2 Hz), 12.01 (2H, s, 2 × OH); 13C NMR (100 MHz, CDCl3) C 193.2, 181.2, 162.5,6, 137.2, 133.6, 124.6, 120.0, 116.0. HREIMS (*m*/*z*): 241.0491 [M+H]^+^.

^1^H NMR and ^13^C NMR data were obtained on a Varian “Mercury 400” spectrometer at 400 and 100 MHz, respectively. Chemical shifts are reported in ppm relative to solvent (CDCl_3_ δH 7.24, δC 77.0). The UV spectrum was recorded in an Agilent 1200 liquid chromatograph with a photodiode array detector. HREIMS was performed on a UPLC 1290 INFINITY II (Agilent Technologies, Santa Clara, CA, USA) coupled with a 6230 TOF LC/MS (Agilent Technologies, Santa Clara, CA, USA).

### 2.3. Cell Culture

Cell culture media were supplemented with penicillin (50 IU/mL) and streptomycin (0.05 mg/mL). Human umbilical endothelial cells (HUVEC) were purchased from Lonza and maintained in EGM-2 medium; following the manufacturers’ instructions, HUVECs were used until passage 9. Breast carcinoma cell line MDA-MB231 was maintained in RPMI supplemented with 10% FBS and glutamine (2 mM). Fibrosarcoma cell line HT1080 was maintained in DMEM containing 4.5 g/L glucose supplemented with 10% FBS and glutamine (2 mM). All cell lines were maintained at 37 °C and in a humidified atmosphere with 5% CO_2_.

### 2.4. Chicken Chorioallantoic Membrane (CAM) Assay

Fertilized chick eggs were purchased by Granja Santa Isabel (Córdoba, Spain) and incubated for three days at 38 °C in a humidified incubator until they were windowed. Five days later, methylcellulose discs with several treatments were implanted on the CAM, and incubated for an additional 48 h. For methylcellulose disc preparation, compounds were resuspended in a 1.2% solution of methylcellulose, and 10 μL drops of this solution were allowed to dry on a Teflon-coated surface in a laminar flow hood. DMSO containing disc was used as a negative control and aeroplysinin-1 (5.9 nmol/methylcellulose disc) was used as a positive control [11]. After incubation, CAM was examined under a stereomicroscope Nikon SMZ 745 T (Nikon, Tokyo, Japan), and photographed with a camera Nikon DS-Ri2 (Nikon, Tokyo, Japan). Results were analyzed by two independent observers. Each condition was scored as positive if a significant reduction or rebound of vessels in the treated area was observed.

### 2.5. Tube Formation on Matrigel by Endothelial Cells

Matrigel (50µL, about 10 mg/mL) was added to each well of a 96-well plate and polymerized at 37 °C for a minimum of 30 min. Then, 2.4 × 10^4^ cells of HUVEC were added in 200 μL of DMEM containing glucose (1 g/L) supplemented with 5% FBS and glutamine (2 mM), with the presence of different compounds, DMSO (negative control), 2 μM staurosporine (positive control) or danthron at different concentrations (2.5, 5, 10 or 25 μM). After 6 h of incubation, cell cultures were photographed with a microscope camera Nikon DS-Ri2 coupled to a Nikon Eclipse Ti microscope (Tokyo, Japan). Closed “tubular” structures were counted using FIJI software 2.1.0/1.53c.

### 2.6. Cell Survival Assay (MTT Assay)

The 3-(4,5-dimethylthiazol-2-yl)-2,5-diphenyltetrazolium bromide (MTT) dye reduction assay was performed in 96-well microplates as previously described [11]. Briefly, 5 × 10^3^ cells for HUVEC, and 2 × 10^3^ for MDA-MB231 and HT1080 were incubated in 96-well-plates in serial dilutions of the desired compound and in quadruplicate. After 72 h of incubation (37 °C, 5% CO_2_ in a humid atmosphere), 10 μL of MTT (5 mg/mL) was added to each well and the plate was further incubated for 4 h. The resulting formazan was dissolved in 0.04 N HCl-2-propanol. Finally, absorbance was read at 550 nm with an Eon Microplate Spectrophotometer and data were collected by Gen5 software 3.0 from Bio-Tek Instruments (Winooski, VT, USA). Half-maximal inhibitory concentration (IC_50_) values were calculated as the concentrations of compound yielding a 50% cell number, using the values obtained for the control condition (cells treated with the vehicle, DMSO) as 100%.

In order to discard and effect the danthron toxicity in the tubulogenesis assay, a modified MTT survival assay was used. HUVECs (2.4 × 10^4^) were treated in 96-well-plates for 6 h (37 °C, 5% CO_2_ in a humid atmosphere) with DMSO (control) or danthron (2.5, 5, 10 and 25 μM). Then, MTT solution was added as described previously and cells were incubated for another 4 h. Thereafter, the resulting formazan was dissolved and absorbance was read at 550 nm.

### 2.7. Proliferation Assay (EdU)

Endothelial and cancer cell proliferation was measured in the presence of danthron using the baseclick EdU Flow Cytometry Kit, according to the manufacturer’s specifications. HUVECs and HT1080 cells were treated for 24 h with 50 μM danthron, DMSO or 3 μM mitomycin C (positive control) and labeled with 10 μM EdU for another 24 h before fixation. For MDA-MB231, cells were treated for 32 h and labeled with EdU solution for another 16 h before fixation. Finally, cells were analyzed in FACS VERSETM flow cytometer (BD Biosciences, Franklin Lakes, NJ, USA). Data were analyzed using BD FACSuite from BD Biosciences and Kaluza Analysis software 2.1 (Beckman and Coulter Life Sciences, San Jose, CA, USA).

### 2.8. Cell Cycle Analysis

Cell cycle analysis was assessed by flow cytometry as previously described [7]. Endothelial and tumor cells were treated with DMSO, 25 or 50 μM danthron and 10 μM 2-methoxyestradiol (2-ME, positive control) for 48 h. Then, cells were harvested, washed with PBS with 1% FBS and HEPES (10 mM) and fixed with 70% ethanol for 1 h on ice. Finally, cells were incubated with 0.1 mg/mL RNAse-A and 40 μg/mL propidium iodide and EDTA 10 mM for 1 h at 37 °C protected from light. Percentages of SubG1, G0/G1 and G2/S/M populations were obtained using a FACS VERSETM cytometer and analyzed using Kaluza Analysis software 2.1 (Beckman and Coulter Life Sciences, Brea, CA, USA).

### 2.9. Hoechst 33258 Staining

Changes in nuclear morphology of endothelial and tumor cells after treatment with danthron were determined by Hoechst 33258 staining. Cells, seeded on gelatin-coat-coverslides, were treated with DMSO, 50 μM danthron or 5 μM 2-ME (positive control) during 24 h. Thereafter, cells on coverslides were washed with PBS and fixed with formalin solution (4%). Finally, coverslides were stained with Hoechst, mounted and photographed using a fluorescence microscope (Nikon Eclipse Ti microscope). Percentages of cells with chromatin condensation were determined in five vision fields from three different experiments.

### 2.10. Zymographic Assay for the Detection of Matrix Metalloproteinase (MMP)

Endothelial (HUVEC) and tumor cells (MDA-MB231 and HT1080) were incubated for 24 h with their respective media without FBS containing 0.1% BSA, 200 KIU/mL aprotinin together with DMSO (for negative control) and several concentrations of danthron (10, 25 and 50 μM). Then, conditioned media were collected, centrifuged at 1000× *g* for 10 min at 4 °C and used for gelatin zymography as previously described [11]. Using a Coulter counter, samples were normalized for equal cell number before being subjected to non-reducing SDS/PAGE with gelatin (1 mg/mL) added to the 10% resolving gel. After electrophoresis, gels were washed twice with 50 mM Tris/HCl, pH 7.4 supplemented with 2% Triton X-100, and incubated for 16 h at 37 °C in a substrate buffer (50 mM Tris/HCl, pH 7.4 supplemented with 1% Triton X-100, 5 mM CaCl_2_, and 0.02% Na_3_N). Finally, gels were stained with Coomassie blue R-250, and photographed with the ChemiDocTM XRS + System (Bio-Rad) using Image LabTM software 6.1. Quantitative analysis was performed using FIJI software.

### 2.11. Invasion Assay

The invasiveness of HUVEC, MDA-MB231 and HT1080 was assayed by using a 24-well clear membrane insert as previously described [33]. Cells were serum starved in medium containing 0.1% BSA for 24 h. Inserts were coated with 100 µL of Matrigel (0.12 mg/mL) and let to dry for 16 h in a laminar flow hood. Then, 5 × 10^4^ HUVECc or 2.5 ×10^4^ tumor cells were added to the inserts in serum-free medium in the absence or presence of danthron. Media with 20% FBS were used as chemoattractants in the lower wells. Serum-free medium was used as the negative control. After 24 h of incubation for HUVEC and HT1080 and 34 h for MDA-MB231, cells were fixed with 4% paraformaldehyde for at least 15 min, dyed with 1% violet crystal (on 2% ethanol) for 20 min and washed with distilled water. Finally, the inserts were photographed and the invading cells were counted with FIJI software and the percentage of invaded cells relative to the positive control was calculated.

### 2.12. Wound-Healing Assay to Determine Cell Migration

The migratory capacity of endothelial and cancer cells in the presence of danthron was assessed using the “wound healing” assay as previously described [7]. Wounded areas were photographed after 0, 4 and 8 h of incubation. The amount of migration was determined by image analysis and normalized with respect to their respective values at time zero by using FIJI software.

### 2.13. Determination of Intracellular Reactive Oxygen Species (ROS) (DCFH-DA Assay)

Intracellular ROS levels were measured using DCFH-DA dye. Then, 6 × 10^4^ cells of HUVECs or 3 × 10^4^ tumor cells (MDA-MB231 and HT1080) were seeded in a 24-well plate and grown until reaching 80–90% confluence. Then, cells were washed with PBS and stained with DCFH-DA prior to their treatment with DMSO (negative control), 50 μM danthron and/or 1 mM hydrogen peroxide (H_2_O_2_). HUVEC and HT1080 cells were stained with 20 μM DCFH-DA during 40 min, while MDA-MB231 cells were stained with 10 μM DCFH-DA for 30 min. Once the treatments were added, measurements were obtained with a fluorescence plate reader every 2 h for 8 h (Ex/em: ~492–495/517–527 nm).

### 2.14. Determination of Sulfhydryl Groups

Cell redox capacity was determined by measuring the disulfide reduction by endothelial and tumor cells as previously described [34]. Cells were treated for 24 or 48 h with tested compounds in 6-well plates (DMSO and 10, 25 or 50 μM of danthron), and they were washed three times with cold PBS supplemented with 1 mM CaCl_2_ and 0.5 mM MgCl_2_. A total of 1 mL of reaction buffer (PBS supplemented with 1 mM CaCl_2_, 0.5 mM MgCl_2_, 5 mM glucose, 0.1 mM lipoic acid and 0.1 mM DTNB) was added to each well and after 1 h of incubation (37 °C, 5% CO_2_ in a humid atmosphere), supernatants were obtained, centrifuged at 13,000× *g* for 5 min and their absorbance at 412 nm was measured. Absorbance was normalized considering the number of cells in each condition and measured using a Coulter counter. As the positive controls, 100 and 50 μM dimetylfumarate (DMF) were used.

### 2.15. Statistical Analysis

Results are expressed as mean ± SD of at least three independent experiments. Statistical significance was determined using the two-sided unpaired Student *t*-test. Values of *p* < 0.05 were considered to be statistically significant. Significance was indicated as follows: **** *p* < 0.0001, *** *p* < 0.001, ** *p* < 0.01, * *p* < 0.05.

## 3. Results

### 3.1. Isolation of Danthron Form the Fermentation Broth of a Marine Fungus

During a blinded screening program of extracts and bioactive compounds isolated from marine organisms, 1,8-Dihydroxyanthraquinone (danthron), produced by the marine fungus, *Chromolaenicola* sp. HL-114-33-R04 was selected by means of its ability to inhibit the in vitro tube formation on Matrigel by endothelial cells. This compound, responsible for the detected antiangiogenic activity of the fungal culture broth, was isolated and the producing fungal strain was identified as follows.

The marine fungus, *Chromolaenicola* sp. HL-114-33-R04, was isolated from the Rhodophyta red algae *Peysonnelia*, collected in a coral reef at Reunion Island in the Indian Ocean. A culture of the strain has been deposited in the Colección Española de Cultivos Tipo at the Universidad de Valencia, Spain. Taxonomical determination was confirmed after a sequencing analysis of the ITS1-5.8S-TS2 ribosomal DNA region. The sequence showed a similarity percentage of 99.27% with the sequence of *Chromolaenicola* sp., a new genus that has been recently described and accommodated in the *Dydimosphaeriaceae* family in *Pleosporales* [35].

The colonies showed a white to light-brown cottony mycelial growth that reached 9 cm in diameter in 15 days at 25 °C on potato dextrose agar with marine salts (20 g/L), in a culture chamber that maintains a humidity of 42%. Fermentation of *Chromolaenicola* sp. (HL-114-33-R04) was carried out in Erlenmeyer flasks, as indicated in the Methods section, and the production of danthron was monitored by HPLC. Fractionation of the fermentation broth crude extract allowed the isolation and chemical elucidation of this anthraquinone derivative, based on spectroscopic data, and their comparison with those of the reported values for danthron [32]. The aqueous solutions of this orange crystalline powder exhibited a significant fluorescence with a broad emission peak reaching a maximum at around λ_em_ 600 nm (λ_exc_ 420 nm), what should be taken into account in order to avoid undesired interferences in those experiments that use fluorescent dyes.

### 3.2. Danthron Inhibits Angiogenesis In Vivo in a Dose-Dependent Manner

The chicken chorioallantoic membrane (CAM), an extraembryonic tissue layer generated by the fusion of the chorion with the vascularized allantoic membrane, has been widely used to evaluate the angiogenic or antiangiogenic activity of molecules. This accessible in vivo model allows the study of the effect of compounds, introduced in the form of small discs, on the CAM neovascularization [36,37]. In the present study, small methylcellulose discs containing different doses of danthron were placed over the CAM and after 2 incubation days the vasculature under and surrounding the disc was evaluated. Pictures displayed in Figure 1C show that danthron reduced not only the ingrowth of new blood vessels in the area covered by the methylcellulose discs, but also, the peripheral vessels around the disc, which grew centrifugally, avoiding the treated area, with an overall decrease in the vascular density. Of note, in the DMSO-treated CAMs (controls), blood vessels formed a dense and spatially oriented branching network composed of vascular structures of progressively smaller diameter as they branch (Figure 1C). As shown in Figure 1B, treatment with danthron caused a dose-dependent antiangiogenic effect, producing an in vivo inhibition of angiogenesis in around 90% of the treated eggs at 20 nmol danthron per CAM. These inhibitory doses are lower than those reported by us and others for other naturally occurring inhibitors of angiogenesis [11,38,39], including the antiangiogenic anthraquinones emodin [40] and aloe emodin [41].

The results obtained with the in vivo CAM assay indicate that danthron is a new antiangiogenic compound, but they give no information on which specific steps of angiogenesis are targeted by this anthraquinone. To obtain additional insights on the features of danthron as an inhibitor of angiogenesis, and to compare them with those exhibited by other antiangiogenic drugs, a set of in vitro assays was used to study its effects on the different steps of angiogenesis.

### 3.3. Danthron Inhibits the Tube Formation on Matrigel by HUVECs at Non Toxic Doses

**Figure 2:**
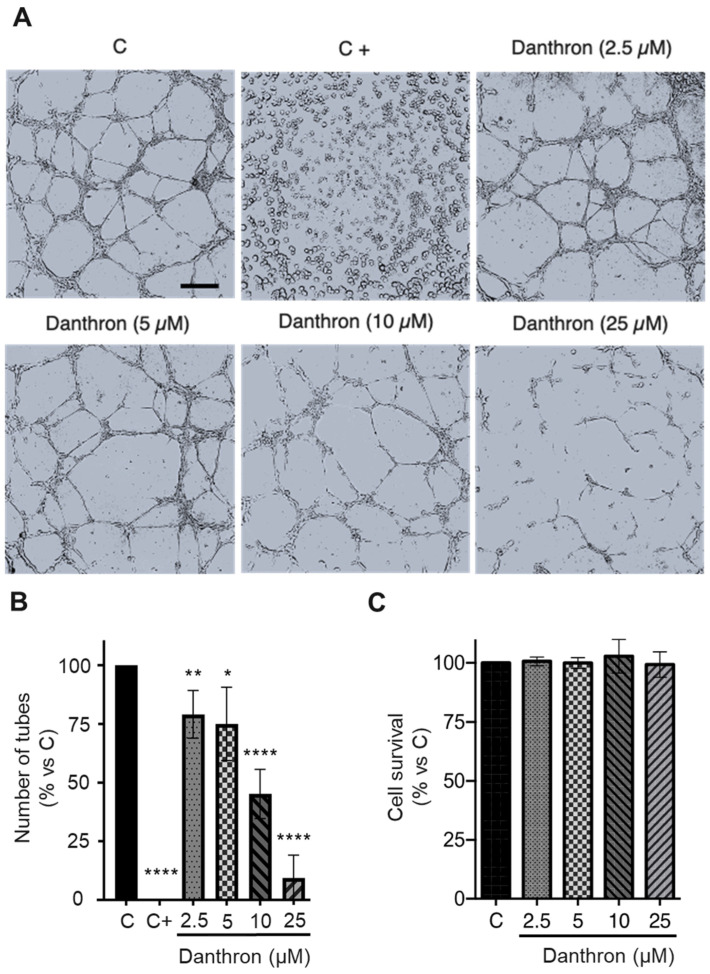
Danthron inhibits the in vitro formation of tubular structures by HUVECs on Matrigel. (**A**) Representative photographs captured 6 h after seeding under an inverted microscope (scale bar = 500 μm). (**B**) Quantitative analysis of the tubular structures formed. Data are expressed as means ± S.D. for three independent experiments; * *p* < 0.05, ** *p* < 0.01, **** *p* < 0.0001 versus control, with the vehicle (DMSO, C). Toluquinol (20 μM) was used as a positive control of inhibition in this assay (C+). (**C**) The MTT assessed the viability of HUVECs under conditions that were similar to those used in this assay.

The organization of endothelial cells in a network of tubes is a crucial event in angiogenesis. The in vitro assay based in the formation of capillary-like structures by endothelial cells on Matrigel has been used by us and others for the high-throughput screening of new inhibitors of this process [19], and, as mentioned before, it was responsible for the detection of the antiangiogenic activity of danthron in our screening program. As shown in Figure 2A,B, danthron exerted a dose-dependent inhibition on the formation of tubular structures by activated HUVECs, being able to almost completely inhibit this process at concentrations that were below the IC_50_ for this compound. A modified MTT assay, in which incubation times with the compound were those used in the tube formation assay, confirmed that this inhibition was exerted at non-toxic concentrations (Figure 2C).

### 3.4. Danthron Inhibits the Growth of Endothelial and Tumor Cells

**Figure 3:**
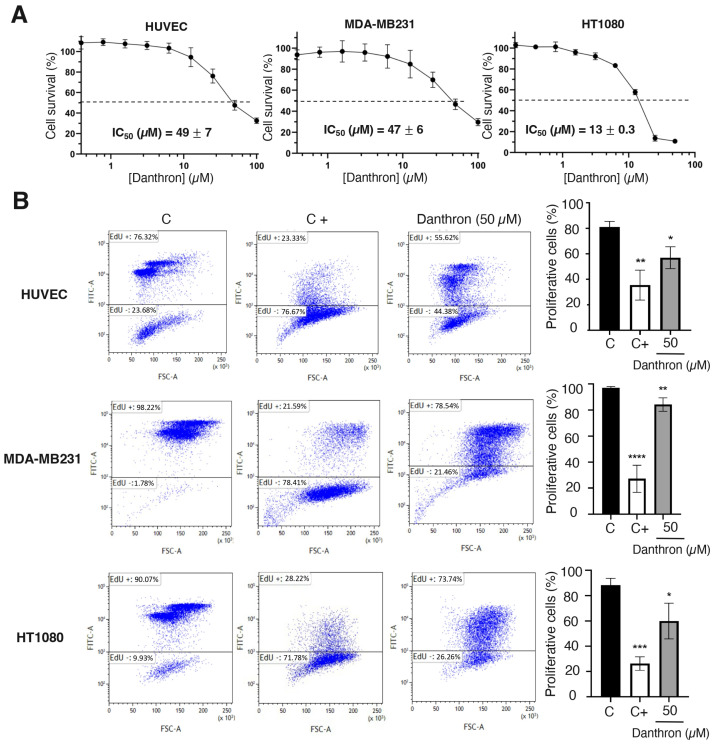
Danthron inhibits endothelial and tumor cell growth and proliferation. (**A**) Effect of danthron on the growth of endothelial and tumor cells. Representative survival curves of proliferative HUVEC, MDA-MB231 and HT1080 cells in presence of increasing concentrations of the compound, performed with quadruplicate samples. Survival was measured after 72 h by adding MTT. IC_50_ values were calculated from dose–response curves as the concentration of compound yielding 50% of control cell survival. IC_50_ values are expressed as means ± S.D. of three independent experiments. (**B**) Effect of danthron on the proliferation of endothelial and tumor cells. (Left) Flow cytometry representative profiles of 24 h EdU-treated HUVEC and HT1080 and 16 h EdU-treated MDA-MB231. Cells were treated for 48 h with DMSO (C), 3 μM mitomycin C (C+) or 50 μM danthron. (Right) Quantitative analysis of EdU+ (proliferative) cells. Data are expressed as means ± S.D. of three independent experiments. * *p* < 0.05, ** *p* < 0.01, *** *p* < 0.001, **** *p* < 0.0001 versus control with DMSO.

Angiogenesis involves the local proliferation of endothelial cells in response to the activation of the angiogenic switch [42]. To evaluate the effect of danthron on the endothelial cell growth, logarithmically proliferating HUVECs were treated with serial dilutions of this compound for 72 h and then the number of viable cells was evaluated by the addition of MTT. A representative dose–response curve is displayed in Figure 3A, where the percentage of viable cells versus the untreated controls in mean of quadruplicates is shown for each concentration of danthron. The half-maximal inhibitory concentration (IC_50_) values, calculated from at least three independent dose–response curves as the concentration of compound yielding 50% of the control cells survival, are also shown in this figure. These IC_50_ values were used to establish the range of concentrations to be used in further studies. As shown in Figure 3A, danthron exerts a similar effect on non-endothelial cells, since the IC_50_ values obtained for human breast carcinoma MDA-MB231 and fibrosarcoma HT1080 cell lines were in the same range of concentrations as that for HUVEC. This is in agreement with previously reported data suggesting a moderate antitumor activity of this compound, based on the inhibition of the in vitro growth of several tumor cell lines [20,21,22,43].

Results from the MTT assay shown in Figure 3A indicated that incubation with danthron decreased the number of viable cells in endothelial and tumor cells, what could be explained, at least in part, by the possible antiproliferative activity of this compound. This hypothesis was confirmed by a cytometric study based on the incorporation into nascent DNA of 5-ethynyl- 2’-deoxyuridine. As shown in Figure 3B, after a 24 h treatment with danthron, a significant reduction in the de novo DNA synthesis by proliferating endothelial and tumor cells was detected.

### 3.5. Danthron Inhibits the Proteolytic and Invasive Capabilities of Endothelial and Tumor Cells

**Figure 4:**
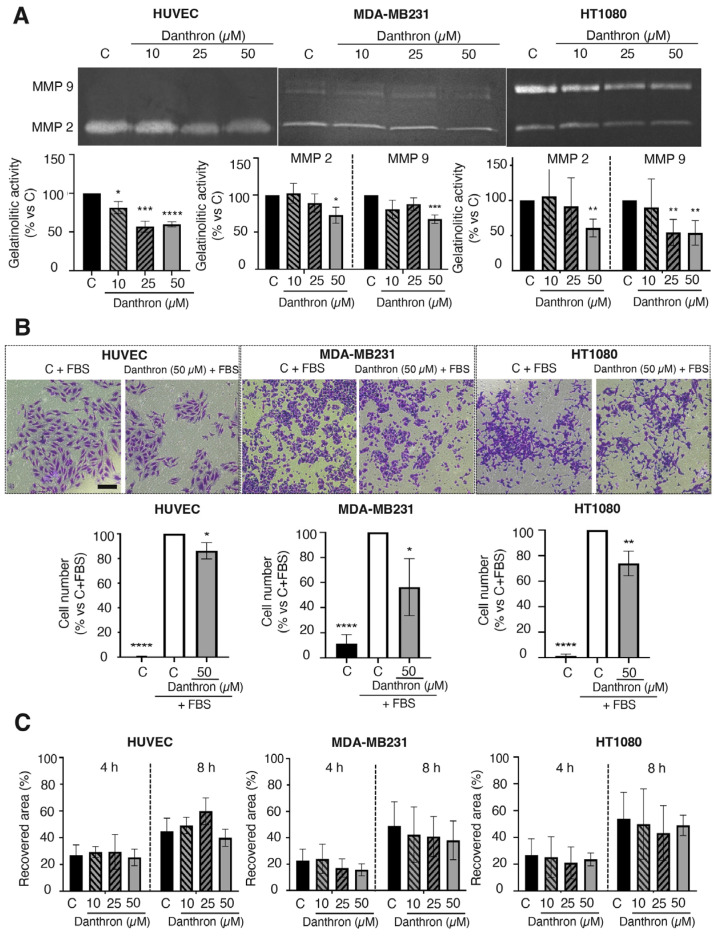
Effect of danthron on the proteolytic, invasive and migratory capabilities of endothelial and tumor cells. (**A**) Effect of danthron on the proteolytic potential of endothelial and tumor cells. Conditioned media from HUVEC, MDA-MB231 and HT1080 cells were analyzed by gelatin zymography. A representative image of the gel (top) and quantification of the zymographic bands of gelatinases (bottom) is illustrated for each cell type. (**B**) Effect of danthron on the in vitro invasive potential of endothelial and tumor cells. Top: representative images of invading cells are illustrated for each cell line (scale bar = 200 μm). Bottom: quantitative analysis of the number of invading cells after a treatment of 24 h for HT1080 and HUVEC, and 34 h for MDA-MB231. (**C**) Effect of danthron on the in vitro migration (wound-healing assay) of endothelial and tumor cells. Quantitative analysis of the recovered area after 4 and 8 h of incubation in the wound-healing assay. Data are expressed as means ± S.D. of three independent experiments. * *p* < 0.05, ** *p* < 0.01, *** *p* < 0.001, **** *p* < 0.0001 versus control with DMSO.

A key step of angiogenesis is the remodeling of the vascular basement membrane and surrounding extracellular matrix by activated endothelial cells [44]. A main role in this process is played by the endothelial MMP-2, which besides degrading several extracellular matrix proteins such as fibronectin, laminin, type I collagen and proteoglycans, is also involved in other functions of endothelial cells [45]. The effect of danthron on the MMP-2 release by HUVECs was evaluated through gelatin zymography. For this, conditioned media of HUVECs, untreated and treated with different concentrations of danthron for 24 h, were subjected to electrophoresis in gelatin-embedded gels and then incubated to allow the gelatin degradation by MMP-2. As shown in Figure 4A (left), the treatment with danthron significantly reduced the MMP-2 secretion by HUVECs in a dose–response manner.

A similar effect was observed when HT1080 and MDA-MB231 tumor cells were incubated in the presence of this compound. As shown in Figure 4A (middle and right), danthron caused a dose-dependent inhibition of the secretion of both MMP-2 and MMP-9 proteases into the medium by tumor cells, indicating that this is not a selective effect on MMP-2, nor is it endothelial specific. These data are in agreement with previous observations by Lin et al. showing that danthron inhibits the proteolytic capability of glioblastoma multiforme GBM 8401 cells [46].

The extracellular matrix degradation by proteases facilitates the endothelial migration and invasion through the tissue towards the proangiogenic stimuli. Since some inhibitors of angiogenesis can reduce the invasive and/or migratory capabilities of endothelial cells, we evaluated the effect of danthron on these competences of activated HUVECs. As shown in Figure 4B, danthron (50 μM) was able to significantly inhibit the invasive potential of endothelial and tumor cells, evidenced by means of the invasion assay on Matrigel-coated transwells. On the contrary, when using the wound-healing migration assay, no significant effect on the migratory capability of endothelial and tumor cells was found (Figure 4C).

### 3.6. Danthron Induces Changes in the Cell Cycle Subpopulations and Apoptosis Only in Tumor Cells

**Figure 5:**
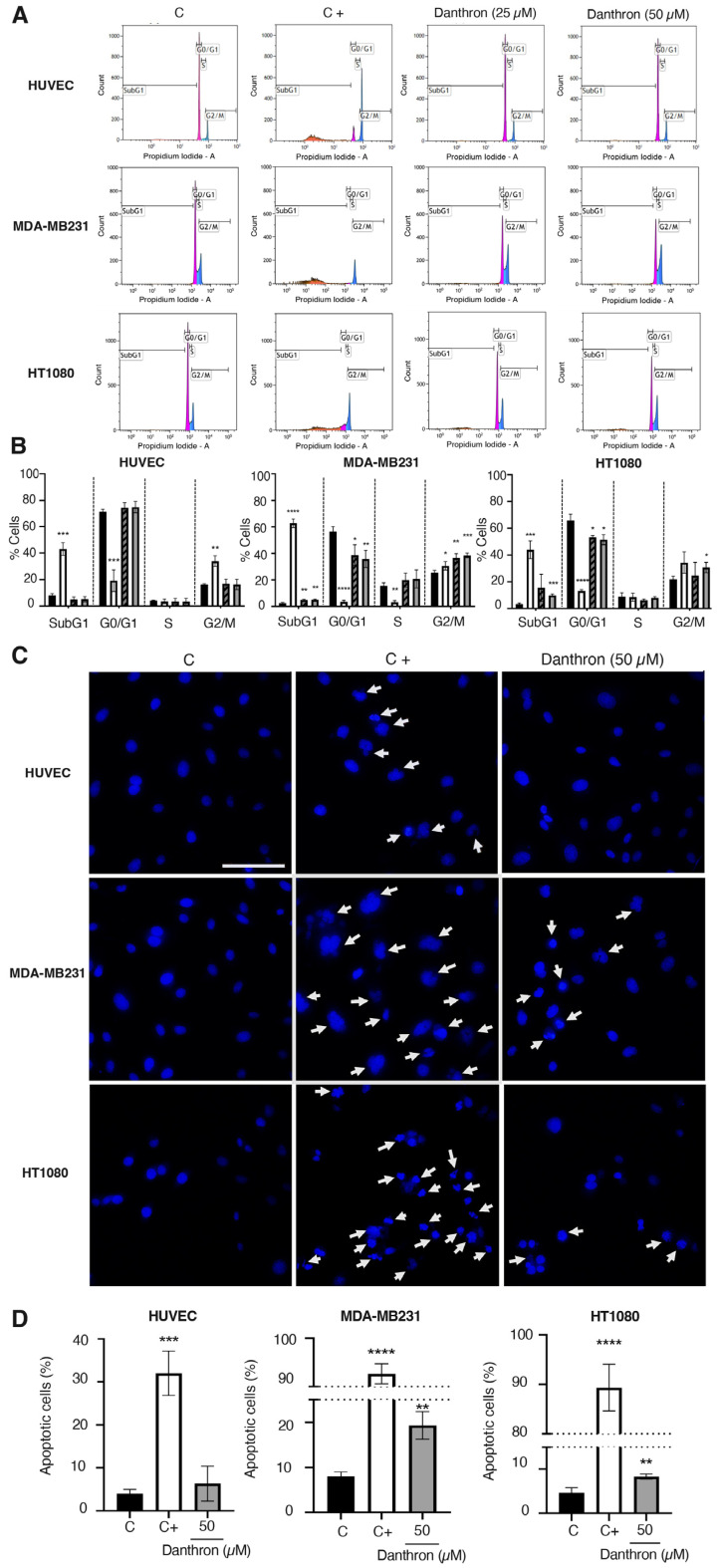
Effect of danthron on cell cycle progression and apoptosis induction. (**A**) Effect of danthron on cell cycle progression. Representative histograms of cell cycle of HUVEC, MDA-MB231 and HT1080 cells treated for 48 h with DMSO (C), 10 μM 2-methoxyestradiol (C+) or the indicated concentrations of danthron, measured by staining with propidium iodide and analyzed by flow cytometry. (**B**) Quantitative analysis of cell cycle experiments. Data are means ± S.D. of three independent experiments. Black columns represent cells treated with DMSO (C), white ones represent cells treated with 5 μM 2-ME (C+), and grey and black columns and grey columns represent cells treated with danthron 25 and 50 μM, respectively. (**C**) Effect of danthron on the cell nuclei morphology of HUVEC, MDA-MB231 and HT1080 cells (Hoechst staining). Representative images are illustrated for each cell line. Cells with condensed chromatin are highlighted by white arrows (scale bar = 50 μm). (**D**) Quantification of the percentage of cells with chromatin condensation. Values are expressed as mean ± S.D. of the cells evaluated in five vision fields from each of three independent experiments. * *p* < 0.05, ** *p* < 0.01, *** *p* < 0.001, **** *p* < 0.0001 versus control with DMSO.

Figure 5A,B summarize the results of the effect of danthron on the cell cycle progression of HUVEC, MDA-MB231 and HT1080 cells, analyzed by flow cytometry. As shown in these figures, incubation of HUVEC with this anthraquinone did not exert any significant differences in the distribution of cells. On the contrary, treatment of MDA-MB231 and HT1080 tumor cells with danthron translated into a significant increase in the subG1 cell population, suggesting a pro-apoptotic activity of this compound on breast and fibrosarcoma tumor cells.

In addition, a significant decrease in the cell population in the G0/G1 phase, and an increase in the population in the S/G2/M phase was observed for the tumor cells but not the endothelial cells (Figure 5A,B). This inversion of peaks in the G0/G1 and G2/M phases indicates that danthron induced a cell cycle arrest in MDA-MB231 and HT1080 tumor cells.

The observation that the treatment with danthron did only exert an increase in the subG1 cell population in the tumor cells led us to further investigate the apoptogenic properties of this compound. For this purpose, the nuclear morphology of the control and treated HUVEC, MDA-MB231 and HT1080 cells was evaluated by using the Hoechst staining. As shown in Figure 5C, danthron (50 μM) induced a significant increase in the number of condensed nuclei in the MDA-MB231 and HT1080 tumor cells, whereas it did not affect the endothelial cells’ chromatin condensation.

This is in agreement with previous findings indicating that the danthron-induced cell death of human brain and gastric cancer cells is closely related to apoptotic death [16,47,48], whereas it discards the suggestion that the induction of endothelial apoptosis could be relevant for the antiangiogenic activity of this compound.

### 3.7. Danthron Reduces Intracellular ROS Production and Increases the Amount of Intracellular Sulfhydryl Groups in Endothelial and Tumor Cells

**Figure 6:**
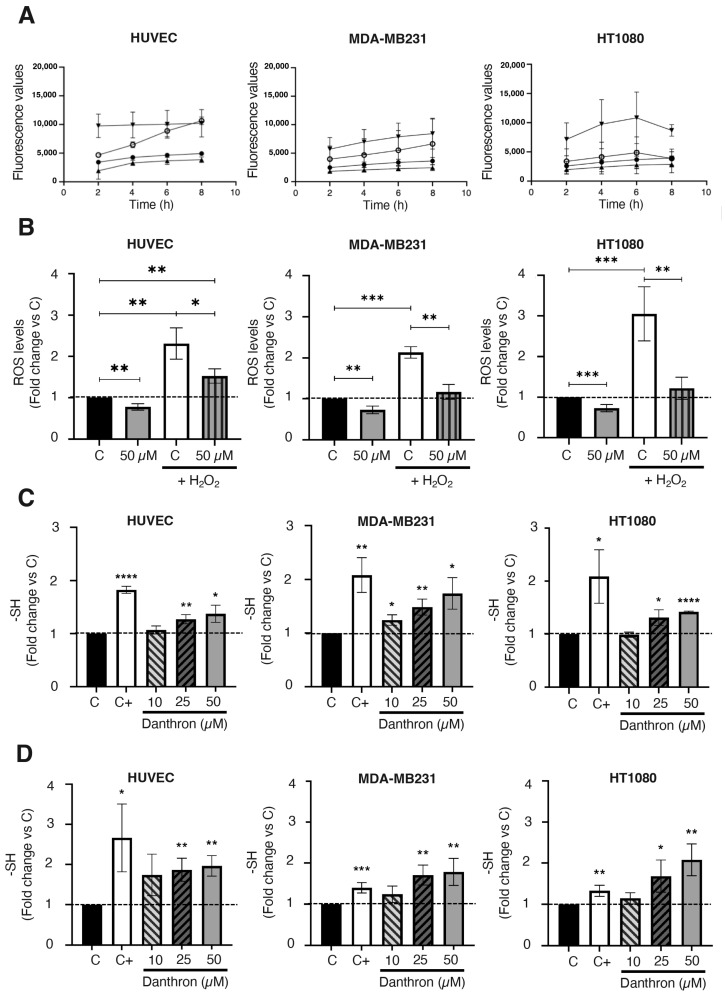
Danthron reduces intracellular ROS production and increases the amount of intracellular sulfhydryl groups in endothelial and tumor cells. (**A**) Effect of danthron on the intracellular ROS generation in HUVEC, MDA-MB231 and HT1080 cells (DCFHDA stain). (**B**) Data show the fluorescence values obtained after staining with DCFH-DA of cells that were treated for 4 h with DMSO or danthron in the presence or absence of 1 mM of hydrogen peroxide. (**C**) Effect of danthron on the amount of intracellular sulfhydryl groups in HUVEC, MDA-MB231 and HT1080 cells after 24 h of treatment. Dimethyl fumarate at a concentration of 100 μM was used as a positive control. (**D**) Effect of danthron on the amount of intracellular sulfhydryl groups in HUVEC, MDA-MB231 and HT1080 cells after 48 h of treatment. Dimethyl fumarate (DMF) at a concentration of 50 μM was used as a positive control. Data are means ± S.D. of three independent experiments (* *p* < 0.05, ** *p* < 0.01, *** *p* < 0.001, **** *p* < 0.0001).

Increases in ROS production seem to be underlying the apoptogenic effect of some drugs [49,50]. In the case of danthron, this issue remains controversial, since some authors have related an increased ROS production with the induction of apoptosis and the antitumoral activity of danthron [16,47,48] while others have proposed a protective effect of danthron against oxidative damage [51]. To shed light on this point, the effect of danthron on intracellular ROS production by HUVEC, MDA-MB231 and HT1080 cells was studied by using the well-established DCFHDA dye assay. In these studies, H_2_O_2_ was also included to further trigger the oxidative stress in those cells. Results presented in Figure 6A,B show that the intracellular ROS production by endothelial and tumor cells significantly decreased after a 4 h treatment with danthron. The antioxidant effect of danthron was more pronounced when ROS production was increased by including H_2_O_2_ in the incubation media. Interestingly, the mentioned decrease in the intracellular ROS production after treatment with danthron inversely correlated with a significant dose-dependent increase in the total amount of sulfhydryl groups found in HUVEC, MDA-MB231 and HT1080 cells that were incubated with this anthraquinone (Figure 6C,D).

## 4. Discussion

The wide pharmacological interest in angiogenesis inhibitors stimulates the search for new drugs that could be used for the treatment of cancer and other angiogenesis-dependent diseases. This study shows that danthron, a hydroxy anthraquinone isolated from the fermentation broth of a marine fungus, is a new inhibitor of angiogenesis, affecting certain key functions of activated endothelial cells, namely, proliferation, proteolytic and invasive capabilities and tube formation.

The antiangiogenic activity of danthron was first detected using the in vitro tubulogenesis assay, showing that this compound inhibits the capillary-like tube formation on Matrigel by HUVECs at non-toxic concentrations that are in the same range or are lower than those required for other previously described inhibitors of angiogenesis, including the natural antiangiogenic anthraquinones damnacanthal, aloe emodin and emodin [8,41,52]. The results obtained with the in vivo CAM assay reinforce the assumption that danthron is a new and potent angiogenesis inhibitor. Additional in vitro assays indicated that this anthraquinone have an inhibitory effect on some key functions of activated endothelial cells, including proliferation, proteolytic and invasive capabilities and tube formation.

The effect exerted by this compound on the growth of activated endothelial cells, evidenced by means of the MTT assay, does not appear to be restricted to this cell type, as the IC_50_ values obtained with this assay in tumor cells ranged in the same order of magnitude. However, some differences were found in the mechanisms that could be responsible for the effect of danthron on cell growth. The results obtained indicate that in tumor cells it could derive from a decrease in proliferation, an arrest in the cell cycle and the induction of apoptosis. This reinforces the previously proposed statement that danthron exhibits a moderate antitumor activity, based on the inhibition of cancer cell proliferation and, in some cases, induction of apoptosis [16,47,48]. In contrast, in endothelial cells, the decrease in cell growth cannot be associated with the induction of apoptosis, nor was any effect on the distribution of cell cycle subpopulations observed after incubation with this compound. According to the data presented here, the inhibition of endothelial cell proliferation could be one of the causes of the observed growth inhibition, although other cell death mechanisms, alternative to apoptosis, cannot be ruled out. These findings suggest differences in the mechanism of action of danthron from that described for the antiangiogenic anthraquinones damnacanthal and emodin, which exhibited a significant apoptogenic activity on endothelial cells that was related to their antiangiogenic properties [8,53].

The putative antimigratory activity of danthron remains a controversial issue, since different results have been reported, depending on the assay and the cell line that were used [46,54,55]. In our studies, using the wound-healing assay, no significant effect of danthron on endothelial or tumor cell migration was observed at the assayed concentrations. In contrast, a significant decrease in the number of invading cells was observed for endothelial and tumor cells in the modified Boyden chamber (transwell) assay. In the case of tumor cells, these results agree with those previously obtained with B16-F10 melanoma cells, what could suggest a putative antimetastatic activity of this compound [54].

Endothelial cells constitutively secrete MMP-2, which is required to trigger tumor angiogenesis in vitro and in vivo [56,57]. A decrease in the production of MMP-2 by endothelial cells has been observed with other angiogenesis inhibitors [8,9,11,13,34,58]. Our data show that incubation with danthron significantly inhibited the MMP-2 secretion by HUVEC. Given that the proteolytic degradation of extracellular matrix components by MMP-2 is critical for angiogenesis, the inhibition of MMP-2 secretion could contribute to the antiangiogenic effect of this anthraquinone. Interestingly, the behavior of HUVECs incubated with danthron differs from that which was observed when bovine aortic endothelial cells (BAEC) were treated with aloe emodin. In that case, despite evidence of an overall balance towards antiproteolysis occurring after incubation with the compound, MMP-2 levels did not decrease, again suggesting that the mechanisms of action of the two antiangiogenic anthraquinones are not identical [41]. Observation that the decrease in the MMP-2 secretion after incubation with danthron occurs in both endothelial and tumor cells, as well as in the second gelatinase produced by tumor cells (MMP-9), suggests that the mechanism of this inhibition of the proteolytic potential is not specific to endothelial cells. In addition, the significant decrease in the production of extracellular matrix metalloproteases, observed when endothelial or tumor cells were treated with danthron, is in agreement with the decrease in their invasive potential, since in the invasion assay that has been employed, the degradation of the extracellular matrix proteins of the Matrigel layer is essential.

Antioxidant properties of danthron are evidenced by the observation that danthron reduces intracellular ROS production and increases the amount of intracellular sulfhydryl groups in endothelial and tumor cells. Our results indicate that, in contrast to what has been proposed for rat glioma and human gastric or glioblastoma cancer cells [16,47,48], danthron did not induce oxidative stress in any of the three cell types used in our assays. Moreover, this compound exerted a protective effect against ROS in endothelial cells, as well as in human breast cancer and fibrosarcoma cells, in agreement with the previous observation that danthron reduced neurotoxicity by various compounds causing oxidative damage in primary cortical cultures [51].

Prior to this study, the antitumor effects of danthron were examined by others in a few cancer cell lines. Those studies pointed to a putative antitumor and antimetastatic activity of this compound, also supported by the data presented here. An additional argument in favor of the use of danthron as a drug candidate derives from exploration by means of several molecular descriptors, including some drug-likeness rules. According to the Muegge pharmacophore point filter, based on the measurement of the topological polar surface area, danthron is clearly a drug-like molecule. Moreover, the surveyed pharmacokinetic behavior of this compound was favorable since it displayed a high gastrointestinal absorption, passively crossed BBB and did not show any potential for P-gp substrate [17]. The drug likeness of danthron, along with its antiangiogenic, antioxidant and antitumor activities reinforce the interest in further studies aimed to unveil the potential of this compound for cancer treatment.

The observation that many natural compounds and herbal drugs possess antiangiogenic, antitumor and antioxidant activities raises the question of whether these activities might be connected to each other [5,6,59,60,61,62,63,64]. Nowadays, there is a body of evidence showing that ROS may function as signaling molecules that modulate several regulatory pathways [65]. Some of them are relevant to tumorigenesis and metastasis and are involved in the control of angiogenesis, invasion, proliferation and survival of tumor cells [65,66]. The role of ROS in the various stages of cancer development has also been related to the chemopreventive potential of antioxidative nutraceuticals [67]. Growing evidence points to a crosstalk between ROS and angiogenesis. ROS may control angiogenesis at different levels, either upstream or downstream in the activation of endothelial cells by angiogenic factors [66]. The mechanism of control for angiogenesis by ROS involves the HIF pathway, Notch signaling, protease production and VEGF transcription through the activation of the NF-kB pathway, among others [68]. A better understanding of the mechanisms of action of danthron could help to elucidate if the antiangiogenic, antioxidative and antitumor activities of this compounds are related.

**Figure 7:**
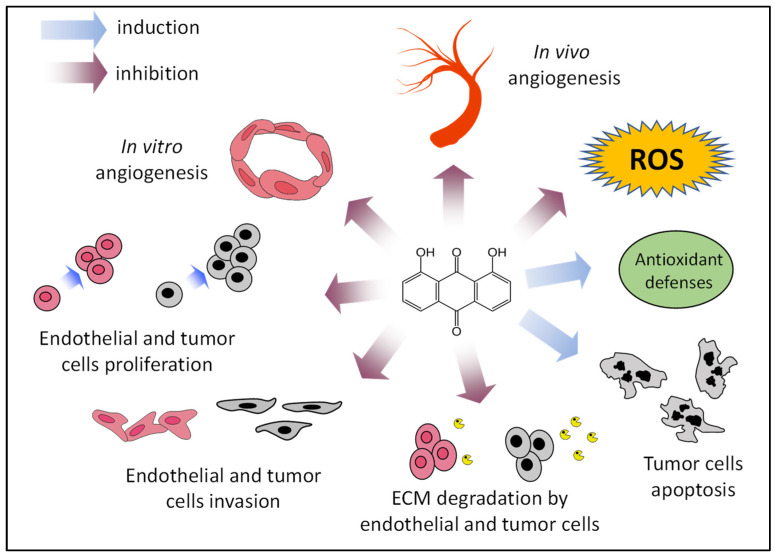
Proposed angiopreventive mechanisms of danthron. ECM, extracellular matrix; ROS, reactive oxygen species.

As mentioned, in addition to the clinical use of inhibitors of angiogenesis, they can also be employed as angiopreventive agents, a new and cost-effective long-term strategy for cancer chemoprevention, aimed at arresting both early primary tumor growth and metastasis by the inhibition of tumor angiogenesis [6]. Figure 7 summarizes some of the presented results, supporting the putative role of danthron as a new angiopreventive agent, based on its antiangiogenic activity, as well as its effects on tumor cells. In addition, findings indicating that the compound could exert an antioxidant effect, although still preliminary, reinforce the idea of the benefits that could be derived from its use in the chemoprevention of cancer and other angiogenesis-dependent diseases. In this line of reasoning, danthron may offer a series of advantages derived from its production by the fermentation of marine fungus. This could ease the use of the purified compound for the secondary or tertiary levels of angioprevention, aimed at delaying onset of the disease in people with a higher susceptibility to suffer from it, as well as increasing the disease-free survival rate in cancer patients. Moreover, the observation that danthron is also present in some plants could suggest the potential of plants such as rhubarb, currently used for other medicinal purposes, in the first level of angioprevention, focused on a wider population.

## 5. Conclusions

Collectively, data from this study show that 1,8- dihydroxy-9,10-anthraquinone (danthron), isolated from the fermentation broth of a marine fungus, inhibits angiogenesis in vitro and in vivo and interferes with certain key functions of activated endothelial cells. Some evidence of the compound’s antitumor and antimetastatic potential, as well as its antioxidant potential, has been shown. The possible existence of a connection between the aforementioned antiangiogenic and antioxidant activities of this compound is a subject that deserves further investigation. These results support a putative role of danthron as a new antiangiogenic drug with potential application to the treatment and the angioprevention of cancer and other angiogenesis-dependent diseases. In-depth studies aimed at unraveling the molecular mechanisms of the antiangiogenic, antitumor and antioxidant activities of danthron are warranted.

## Data Availability

The data are contained within the article.
